# Supplementary material for: EnzML: multi-label prediction of enzyme classes using InterPro signatures
Source: BMC Bioinformatics. 2012 Apr 25;13:61. doi: 10.1186/1471-2105-13-61 (PMC3483700; doi:10.1186/1471-2105-13-61)
Supplement: Addtional file 5 — The Java code to format the data files, evaluate and predict. The file enzml_java_code.tar.gz contains the Java code used to format database data to ARFF and XML formats, to execute cross and train-test (jackknife) evaluations and to record evaluation results to database. More information is included in the readme.txt file and the Javadoc files. The code can be used with a MySQL database. To use a different database software, other JDBC drivers might be required. [file 1471-2105-13-61-S5.gz › java_code/enzml2011/doc/uk/ac/ed/inf/enzml/mulan/learn/EvaluatorNoAUC.html]

EvaluatorNoAUC


---


|  |  |  |
| --- | --- | --- |
| **Overview** \| **Package** \| **Class** \| **Use** \| **Tree** \| **Deprecated** \| **Index** \| **Help** | |  |
| **PREV CLASS**   **NEXT CLASS** | **FRAMES**    **NO FRAMES**     **All Classes** |  |
| SUMMARY: NESTED \| FIELD \| CONSTR \| METHOD | DETAIL: FIELD \| CONSTR \| METHOD |  |


---


## uk.ac.ed.inf.enzml.mulan.learn Class EvaluatorNoAUC

```
java.lang.Object
  mulan.evaluation.Evaluator
      uk.ac.ed.inf.enzml.mulan.learn.EvaluatorNoAUC
```

**Direct Known Subclasses:**: MulanCrossEvaluator

---

``` public class EvaluatorNoAUC extends mulan.evaluation.Evaluator ```

Prepares the evaluation measures taking into consideration the dataset size.
MicroAUC and MacroAUC are \*not\* calculated for datasets > n instances.

**Version:**
:   7 Apr 2011

**Author:**
:   Luna De Ferrari luna.deferrari-at-ed.ac.uk

---

**Constructor Summary**

`EvaluatorNoAUC()`


| **Method Summary** | |
| --- | --- |
| `mulan.evaluation.Evaluation` | `evaluate(mulan.classifier.MultiLabelLearner learner, mulan.data.MultiLabelInstances data)`             Evaluates a `MultiLabelLearner` on given test data set. |

**Methods inherited from class mulan.evaluation.Evaluator**

`crossValidate, crossValidate, evaluate, setSeed, setStrict`

**Methods inherited from class java.lang.Object**

`equals, getClass, hashCode, notify, notifyAll, toString, wait, wait, wait`

**Constructor Detail**

### EvaluatorNoAUC

```
public EvaluatorNoAUC()
```


**Method Detail**

### evaluate

```
public mulan.evaluation.Evaluation evaluate(mulan.classifier.MultiLabelLearner learner,
                                            mulan.data.MultiLabelInstances data)
                                     throws java.lang.IllegalArgumentException,
                                            java.lang.Exception
```

:   Evaluates a `MultiLabelLearner` on given test data set.

    :   **Overrides:**: `evaluate` in class `mulan.evaluation.Evaluator`
    :   **Parameters:**: `learner` - the learner to be evaluated: `data` - the data set for evaluation **Returns:**: the evaluation result **Throws:**: `java.lang.IllegalArgumentException` - if either of input parameters is null.: `java.lang.Exception`


---


|  |  |  |
| --- | --- | --- |
| **Overview** \| **Package** \| **Class** \| **Use** \| **Tree** \| **Deprecated** \| **Index** \| **Help** | |  |
| **PREV CLASS**   **NEXT CLASS** | **FRAMES**    **NO FRAMES**     **All Classes** |  |
| SUMMARY: NESTED \| FIELD \| CONSTR \| METHOD | DETAIL: FIELD \| CONSTR \| METHOD |  |


---
